# The Influence of Heat Treatment on the Microstructure and Properties of a Cu-Bearing Ultra-Low Carbon Steel

**DOI:** 10.3390/ma17123031

**Published:** 2024-06-20

**Authors:** Weina Zhang, Zhanjie Gao, Huimin Zhang, Hao Wei, Zejin Chen, Wenying Xue, Zhenyu Liu

**Affiliations:** State Key Laboratory of Rolling and Automation, Northeastern University, Shenyang 110819, China

**Keywords:** Cu-bearing ultra-low carbon steel, heat treatment, Cu precipitation, microstructure, mechanical property

## Abstract

This study reveals the relationship between the Cu precipitates and mechanical properties of a Cu-baring ultra-low carbon steel after two-phase zone quenching and tempering at 923 K for 0.5–2.5 h. The tensile and microstructural properties were investigated as a function of heat treatment time. The contribution of the precipitation-strengthening mechanism to yield strength was calculated. The size, morphology, and distribution of the precipitated particles were observed using TEM. As the heat treatment time increased, the strength gradually decreased and then remained stable, and the elongation gradually increased and then remained stable. Additionally, the contributions of each strengthening mechanism to the yield strength under different heat treatments were 117, 107, 102, and 89 MPa, respectively. The size and quantity of the precipitates increased with the increase in heat treatment time. After tempering for more than 2 h, the precipitates continued to coarsen, but their quantity decreased. The precipitated Cu had a 3R structure with a length of approximately 17.1 nm and a width of approximately 9.7 nm, with no twinning inside. The stacking order was ABC/ABC. The stable Cu precipitation structure was FCC, maintaining a K-S orientation relationship $\left(1\bar{1}1\right)$_FCC Cu_ //(0 1 1) _α_, $\left[\bar{1}10\right]$_FCC Cu_//$[1\bar{1}1$] _α_.

## 1. Introduction

Ultra-low carbon hull structure steels are designed by utilizing the precipitation strengthening effect of Cu to compensate for the strength loss caused by the ultra-low carbon content, combining it with alloy elements such as Ni, Mn, Cr to obtain high strength, high toughness, and good welding performance [1,2,3,4,5]. Compared with traditional ship structural steel, the content of other alloy elements, except for Cu, is lower, and its carbon content and carbon equivalent are also much lower than the same strength grade steel [6,7,8].

Research has shown that the peak in hardness, yield strength, and tensile strength corresponded to the formation of a large number of coherent Cu precipitates at an aging temperature of 723 K in ultra-low carbon steels [7]. Therefore, it is of great importance to develop new high-strength hull structural steel with an excellent comprehensive performance and low costs.

In recent years, the conventional quench and temper heat treatment (QT) has been widely used to improve the strength properties of hull structure steel, such as HSLA steel [9]. In addition, in order to obtain excellent comprehensive mechanical properties, a critical heat treatment is introduced in the QT process, which is to fully austenitize and quench before heating to the critical heat treatment range between A_C1_ and A_C3_ and then rapidly cool to room temperature. Under the conditions of critical heat treatment (IQT), the microstructure characteristics of steel, the size and quantity of residual austenite, and the combined effect of the precipitated phase particles and alloy elements usually result in a significant combination of steel strength and toughness [10,11]. Therefore, it is necessary to study the effect of the heat treatment process on the microstructure of new ship plate steel.

Precipitation strengthening is mainly caused by the interaction between the precipitate phase and dislocations in the matrix. Its effect is significantly affected by factors such as the size, shape, distribution of the precipitate phase, as well as the strain rate and temperature of the material [12,13,14]. A study on the microstructure and strengthening behavior of Cu containing non-oriented silicon steel showed that Cu precipitation significantly increased the yield strength of the material by about 207 MPa without affecting the magnetic properties [15]. Another study also showed that when Cu precipitates form nanoclusters, the yield strength of the material can increase by 300–400 MPa [16].

Goodman et al. [17,18] found that the hardness of the Fe Cu alloy reaches its maximum when the number and density of BCC FeCu (body-centered cubic) clusters precipitate, and it transforms into FCC Cu (face-centered cubic) structures when the precipitated particles exceed 5 nm. P. J. Othen et al. [19] first proposed the evolution process of the Cu precipitation crystal structure, with a specific evolution pattern of BCC Cu → 9R Cu → 3R Cu → FCC Cu. R. Monzen [20] proposed different crystal structure evolution processes, with the evolution sequence being BCC Cu → 9R Cu → FCT Cu → FCC Cu. P. J. Othen’s research found that the 3R Cu structure does not have twinning but has actually a twisted FCC structure, and its orientation relationship with the matrix is similar to the K-S relationship. In addition, 3R Cu is considered a metastable structure, which undergoes lattice relaxation to transform into a stable FCC Cu structure after the Cu rich phase grows to a certain extent and maintains a K-S relationship with the matrix [21]. On the other hand, R. Monzen and other scholars believe that the structure of 3R Cu was transformed into two variants, FCT Cu and FCC Cu, through the rotation of the lattice plane and changes in the interplanar spacing [22,23,24]. And Y. U. Heng et al. [25] denied the existence of the 3R Cu structure, believing that 9R Cu is a distorted FCC structure that can directly transform into a twin FCC structure by eliminating stacking faults, bypassing the intermediate transition stage from 9R Cu to 3R Cu. There is currently no consensus in the academic community on the evolution sequence of the crystal structure of Cu precipitates, and further research is needed to explore it in depth.

This article introduces the influence of heat treatment on the Cu precipitates and mechanical properties of a Cu-bearing ultra-low carbon steel after two-phase zone quenching and tempering. The contribution of the precipitation-strengthening mechanism in yielding strength was calculated. The evolution of Cu precipitation was investigated using TEM (FEI Tecnai G^2^ F20, FEI, Inc., Valley City, ND, USA) and HRTEM (high-resolution transmission electron microscope). This helps to deepen the understanding of the precipitation strengthening and evolution mechanism of the Cu-bearing ultra-low carbon. 

## 2. Materials and Method

**Figure 1:**
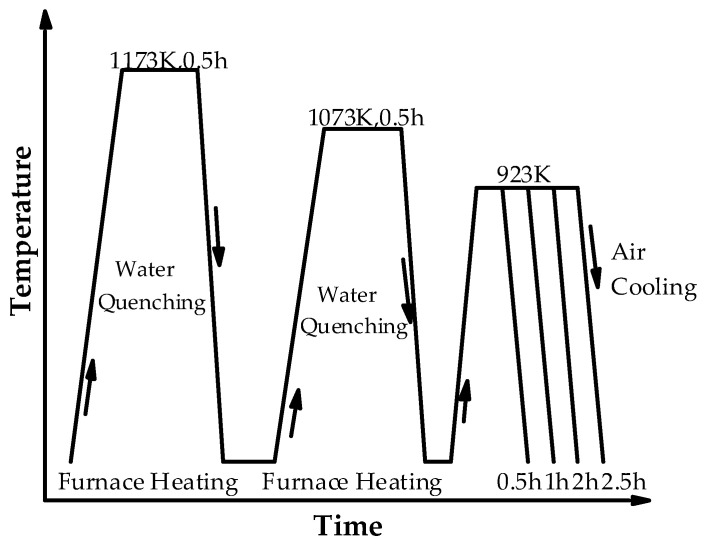
Heat treatment process of the experimental steel.

In the current study, the experimental steel containing 0.02 wt% C and 1.52 wt% Cu (specific alloy composition was listed in Table 1) was produced using a vacuum induction furnace under an argon atmosphere and then cast into an ingot weighing 25 kg. The tested steels were cast into 25 kg ingots by vacuum melting and forged into billets with a size of 80 × 80 × 120 mm^3^. The ingot was homogenized at 1473 K for 2 h and then forged into a thickness of 80 mm. After eliminating the surface defects, the ingot was hot-rolled to plates with a thickness of 10 mm (with an initial rolling temperature of 1423 K and finishing temperature greater than 1073 K, and then water-quenched). The heat treatment process diagram is shown in Figure 1. The experiment adopts a two-phase zone quenching + tempering (IQT) process. The quenching temperature selected 1173 K, held for 0.5 h, followed by water cooling to room temperature. Then, it was reheated at 1073 K and held for 0.5 h, followed by water cooling to room temperature. The tempering processes were held at different tempering times, i.e., 0.5 h, 1 h, 2 h and 2.5 h, at 923 K, followed by water-quenching to reach room temperature.

These experimental specimens were mechanically ground to #2000. Before conducting microstructure observations, all specimens were electrochemically polished in a 12.5% perchlorate acid solution at 30 V for 30 s to remove the residual stress. The microstructure and Cu precipitation character distribution were characterized by using focused ion beam field emission scanning electron microscopy (Zeiss, ULTRA55, Oberkochen, Germany) and field emission transmission electron microscopy (FEI Tecnai G^2^ F20). SEM samples were prepared and etched with a 2% nital solution. The TEM samples were prepared by cutting thin slices and burnishing them using sandpapers, followed by an electropolisher in an electrolyte mixture containing 92% ethyl alcohol and 8% perchloric acid. To evaluate the mechanical properties of the tested steel, the tensile test was conducted using a CMT-5105 testing machine (SANS, Shenzhen, China). The tensile test of the sample after heat treatment was carried out in accordance with GB/T 228.1-2010 [26]. The diameter of the sample was 6 mm, the standard distance was 30 mm parallel to the rolling direction, and the tensile rate was 1 mm/min. 

## 3. Results

### 3.1. Tensile Properties

**Figure 2:**
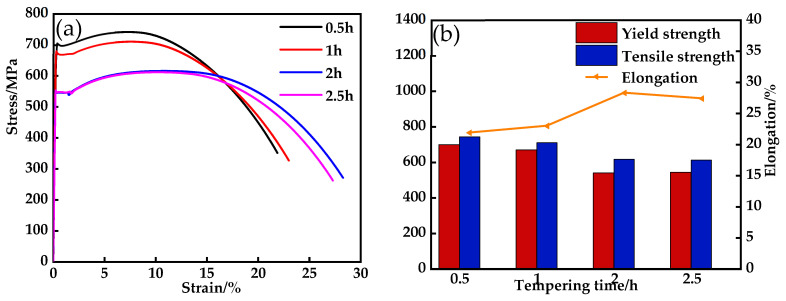
Tensile properties of the experimental steel under different heat treatment conditions: (**a**) typical stretch curve; (**b**) tensile properties change trend.

The tensile curves of the experimental steels at different heat treatment times are shown in Figure 2a, and the statistics of yield strength, tensile strength, and elongation are shown in Figure 2b. In the IQT process, as the extension of the tempering time increased, the strength gradually decreased and then remained stable, and the elongation gradually increased and then remained stable. When the tempering time was 0.5 h, the yield strength was 696 MPa, the tensile strength was 741 MPa, and the elongation was 21.9%. When the tempering time was 1 h, the yield strength decreased to 667 MPa, the tensile strength was 709 MPa, and the elongation was 23%. At this point, the tempered bainite underwent restitution and recrystallization. Continuing the tempering for 2 h, the yield strength was further reduced to 537 MPa, the tensile strength to 615 MPa, and the elongation was significantly increased to 28.3%. After 2.5 h, the yield strength was 542 MPa, the tensile strength was 615 MPa, and elongation was slightly reduced to 27.4%. In the tempering time between 2 and 2.5 h, the precipitation-strengthening effect and the tempering-softening effect reached an equilibrium state. 

### 3.2. Microstructural Characterization by SEM

**Figure 3:**
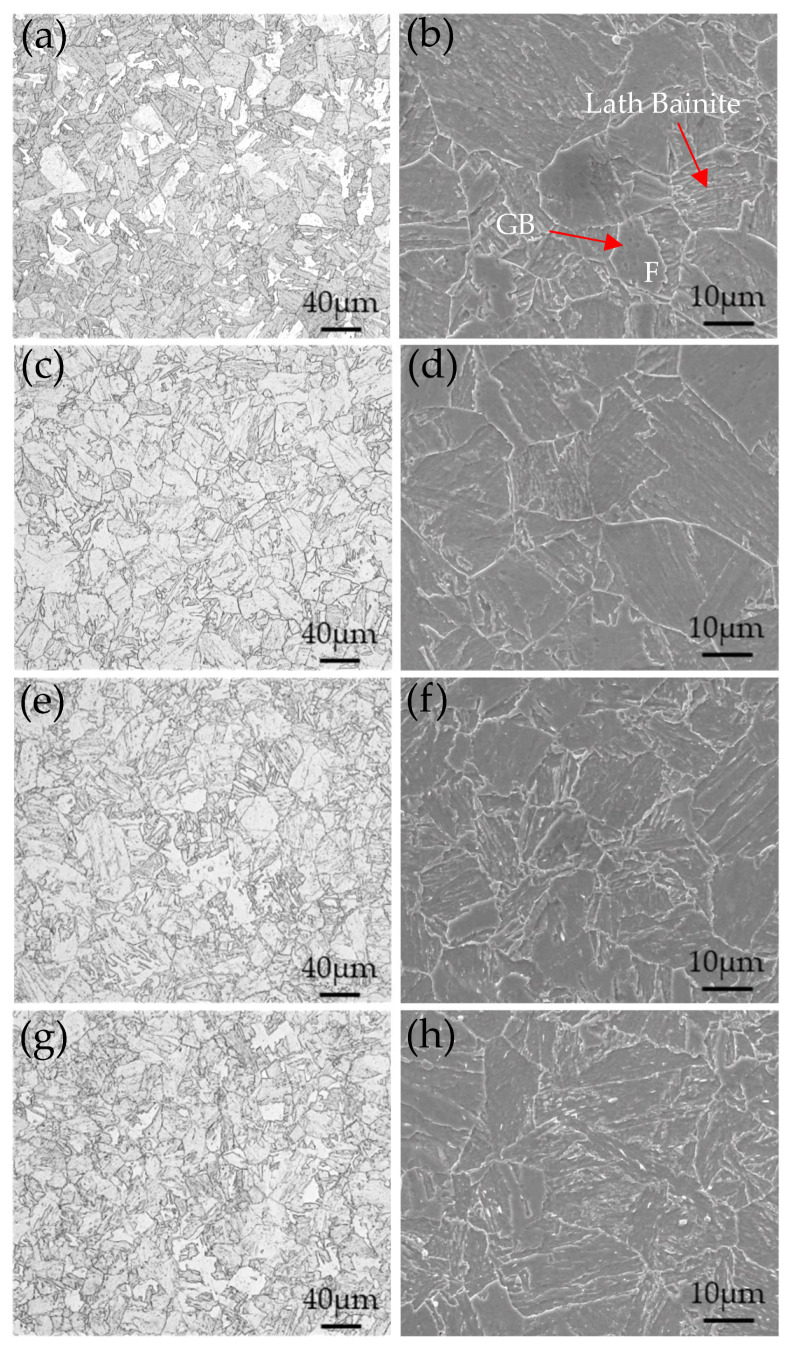
OM and SEM images of microstructure under different heat treatment conditions: (**a**,**b**) 0.5 h, (**c**,**d**) 1 h, (**e**,**f**) 2 h, and (**g**,**h**) 2.5 h.

Figure 3 shows the optical microstructure and SEM image of the experimental steel after critical temperature quenching and tempering treatment. It could be observed that it consists mainly of tempered bainite by its optical microstructure. The purpose of critical temperature heating is to refine the existing microstructure without complete transformation to austenite. The partial reversion and refinement of the existing bainite microstructure occur, which improves the plasticity and toughness of the steel and, at the same time, reduces the internal stresses caused by quenching. There were lath bainite, granular bainite, and a small amount of ferrite in the structure with a tempering time of 0.5 h by SEM, as shown by the arrow in Figure 3b. The tempering time exceeded 1 h, and there were no significant changes in the bainite matrix. As the tempering time increased, bainite recrystallization occurred, the number of lath bainite decreased significantly, and bainite recovery and recrystallization occurred. The tempering time reached 2.5 h, the amount of bainite recrystallization increased, and the structure changed from lath to equiaxed crystal.

### 3.3. Microstructural Characterization by TEM

**Figure 4:**
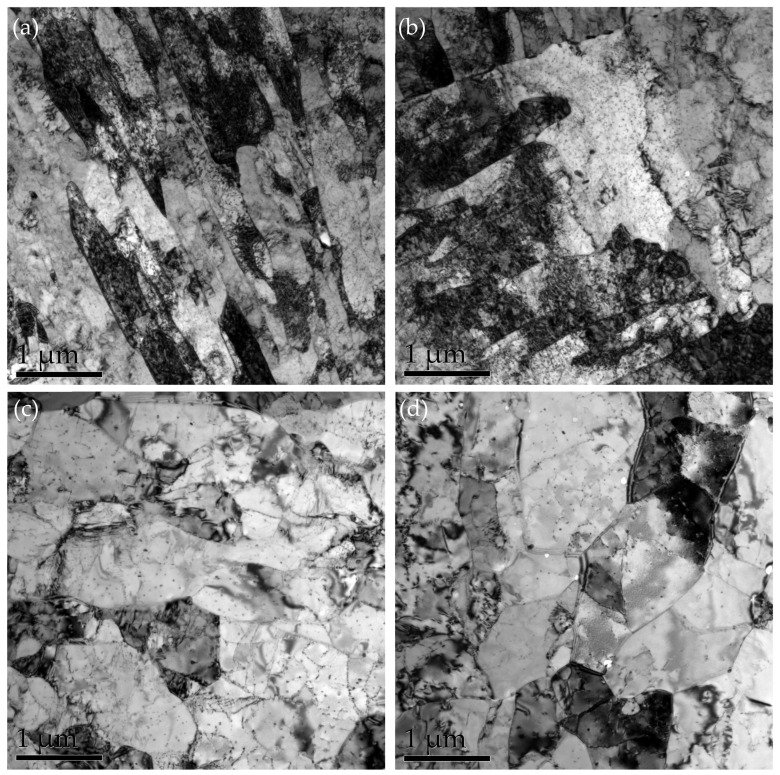
TEM images of microstructure under different heat treatment conditions: (**a**) 0.5 h, (**b**) 1 h, (**c**) 2 h, and (**d**) 2.5 h.

Figure 4 shows the TEM image of the experimental steel after critical temperature quenching and tempering treatment. Figure 4a shows the microstructure image after tempering for 0.5 h, and the height distortion of the supersaturated bainite produced by quenching begins to decrease. Carbon and other alloying elements began to precipitate out of the supersaturated bainite solid solution, forming fine carbides and precipitates that further strengthened the material. With the tempering time lasting 1 h, the tempered bainite began to recover, the boundaries of its laths became unclear, the dislocation density in the crystal decreased, and sub grains were formed. When the tempering time was 2 h, the tempered bainite and recrystallization phenomenon reappeared, and the new dislocation-free or low dislocation density grains would grow out of the original deformation of the grain, eliminating the original grain distortion and dislocation structure. The number of precipitates in the microstructure was reduced and the size increased after tempering for 2.5 h.

**Figure 5:**
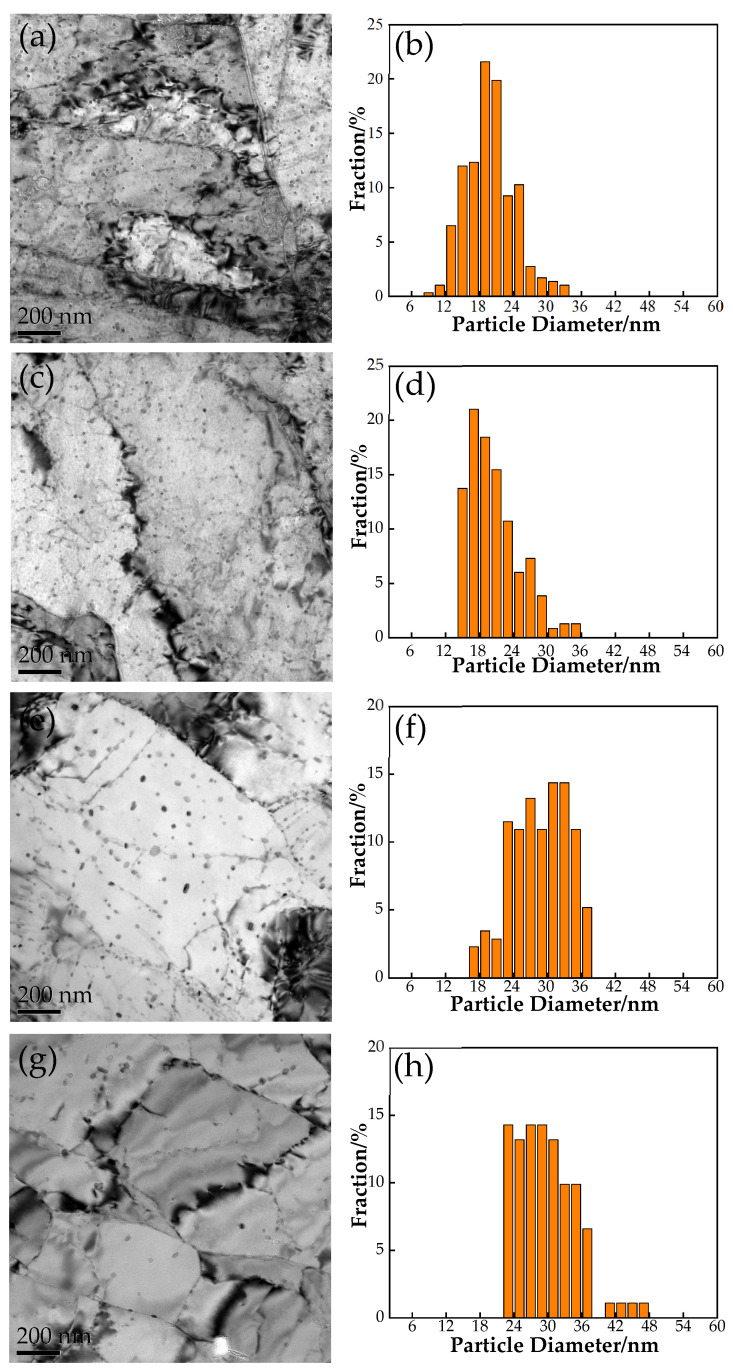
Statistical analysis of precipitation particle size under different heat treatment conditions: (**a**,**b**) 0.5 h, (**c**,**d**) 1 h, (**e**,**f**) 2 h, and (**g**,**h**) 2.5 h.

The precipitation phases of the experimental steel in different states were captured and statistically analyzed using transmission electron microscopy technology. The results of heat treatment time on the size, structure, and distribution of nano-precipitates are shown in Figure 5. It could be observed that the size of the precipitated phase increased with increasing tempering time. When the tempering time was 0.5 h, the size distribution of the precipitated particles was mainly 12–26 nm, with an average particle size of about 16.8 nm and a density of 2.08 × 10^21^ m^−3^. As the tempering time increased to 1 h, the size distribution of precipitated particles was mainly 14–24 nm, with an average particle size of about 17.1 nm and a density of 1.8 × 10^21^ m^−3^. It can be seen that as time increased, the size of the precipitates coarsened, but the density decreased. The size distribution of the precipitated particles was mainly 16–28 nm, with an average particle size of about 25.9 nm and a density of 8.42 × 10^20^ m^−3^, when the tempering time was 2 h. Compared to 1 h, more Cu elements tended to aggregate and precipitate, resulting in coarsening. At this stage, more nano-Cu precipitation was generated instead of coarsening. Cu precipitation occurred in unstable structures. When the tempering time was 2.5 h, the size distribution of the precipitated particles was mainly 22–36 nm, with an average particle size of about 30.6 nm and a density of 6.03 × 10^20^ m^−3^. The number of precipitated phases decreased, and the size increased.

**Figure 6:**
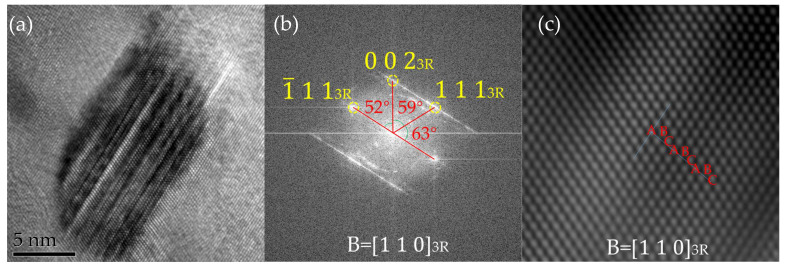
High-resolution image analysis of 3R Cu at 650 °C for 2 h. (**a**) 3R Cu image, (**b**) the FFT pattern of (**a**), (**c**) IFFT image after filtrating of 3R Cu.

The crystal structure of the nano-precipitates was studied using high-resolution electron microscopy technology. Figure 6 shows the experimental steel tempered at 923 K for 2 h after critical heat treatment, taken in the $\left[1\bar{1}1\right]$axis of the Fe matrix. Its length and width were approximately 17.1 nm and 9.7 nm, respectively. As shown in Figure 6a, there were no twin crystals inside 3R Cu, and the appearance of these stripes was significantly different from the regularity and clarity of 9R Cu. The stripes were irregular and occasionally merged or disappeared. Figure 6b shows the fast Fourier transform image of Figure 6a and the calibration results of the 3R Cu structure. The stacking order was ABC/ABC. Among them, d _(111)_ = 0.204 nm, d _(002)_ = 0.176 nm, the angle between (111) _3R_ and $\left(\bar{1}11\right)$_3R_ was about 63°, the angle between (111) _3R_ and (002) _3R_ was about 59°, and the angle between $(\bar{1}11$) _3R_ and (002) _3R_ was about 52°.

**Figure 7:**
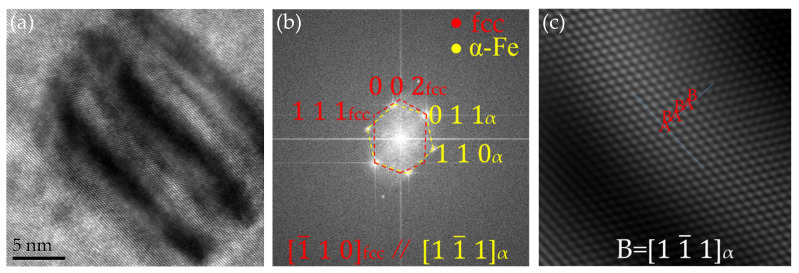
HRTEM analysis of ellipsoidal FCC Cu at 650 °C for 2 h. (**a**) FCC Cu image, (**b**) the FFT pattern of (**a**), (**c**) IFFT image after filtrating of matrix.

Figure 7 shows the stable FCC structure formed by the precipitated particles at 923 K for 2 h. The electron beam was parallel to the $\left[1\bar{1}1\right]$_α_ axis. When incident in the direction, a high-resolution lattice stripe pattern of a rod-shaped Cu rich phase and its corresponding fast Fourier transform image were obtained. As shown in Figure 6a, the long axis of this Cu rich phase was approximately 25 nm, while the length of the short axis was approximately 17.34 nm. Its contrast shows the presence of coarse Moiré fringes, indicating that it is transformed from a twinned 9R Cu structure to a de-twinned FCC Cu structure. The growth direction of the FCC Cu precipitate phase was parallel to the $\left(1\bar{1}1\right)$FCC plane, resulting in its final growth from spherical to rod-shaped. Figure 6b shows the Fourier transform image and diffraction spot calibration results of Figure 6a. The results indicate that nano-precipitation had an FCC Cu structure with no twinning inside. The crystal plane spacing corresponding to the diffraction spots of (002) _FCC Cu_ and (111) _FCC Cu_ are 0.1823 nm and 0.2064 nm, respectively. The calculated lattice constant a = 0.3646 nm, which was basically consistent with the lattice constant of pure Cu. In this type of FCC Cu structure without twinning, although the size of the precipitated phase may further increase, its crystal structure remains unchanged. Therefore, it can be considered as a stable FCC Cu structure. The FCC Cu and matrices were completely different in the lattice and maintained a K-S orientation relationship as follows: (1$\bar{1}1)$_FCC Cu_//(0 1 1) _α_, $\left[\bar{1}10\right]$_FCC Cu_//$\left[1\bar{1}1\right]$ _α_.

## 4. Discussion

### 4.1. Influence of Heat Treatment on Precipitation Strengthening

The strengthening effect of steel mainly comes from mechanisms such as solid solution strengthening, precipitation strengthening, fine-grained strengthening, phase transformation strengthening, and dislocation strengthening. The strengthening effect of the steel used in the experiment is usually not directly determined by a single mechanism but the result of the combined action of multiple mechanisms. This study focuses on investigating the effect of precipitation strengthening on the strength of the experimental steel. In the process of metal deformation, precipitates can hinder the movement of dislocations, forcing them to bypass or cut through the second-phase particles. This process consumes energy and, therefore, requires an increase in external stress to achieve metal strengthening. Precipitation strengthening is an extremely effective strengthening method in microalloyed high-strength steel, and even a small amount of microalloying elements can significantly enhance the strength of experimental steel. At the same time, precipitates also play a key role in grain refinement. In particular, Cu-rich nano-precipitates play an important role in the strengthening process. Due to the significant changes in the size and distribution of precipitates in the experimental steel, for simplicity, this study used the Russell–Brown model to calculate the precipitation-strengthening effect. In this model, particles have attractive interactions with dislocations and play a role in fixing dislocations, resulting in a strengthening effect. For weak particles, such as Cu-rich nano-precipitates, dislocations will penetrate them, and the corresponding strengthening effect can be evaluated using the following formulae proposed by Russell and Brown to enhance precipitation [27,28]:

(1) $$F_{m}=M\frac{Gb}{L}{\left[1-{\left(\frac{E_{p}}{E_{m}}\right)}^{2}\right]}^{\frac{3}{4}};\;\sin^{-1}\left(\frac{E_{p}}{E_{m}}\right)\geq 50{}^{\circ}$$

(2) $$\frac{E_{p}}{E_{m}}=\frac{E_{p}^{\infty }\log\frac{R}{r_{in}}}{E_{m}^{\infty }\log\frac{r_{out}}{r_{in}}}+\frac{\log\frac{r_{out}}{R}}{\log\frac{r_{out}}{r_{in}}}$$

(3) $$L=0.866{\left(RN\right)}^{-1/2}$$

where *M* = 3 represents the Taylor factor, *b* = 0.25 nm refers to the Burgers vector, and *G* = 80 GPa is the shear modulus of the Fe matrix. *E_p_* and *E_m_* are the energies of the precipitate and dislocation lines in the matrix, respectively. L represents the average distance between the particles on the slip plane. The ratio of *E_p_*/*E_m_* is related to the radius R of the particle, where *r_in_* = 2.5 b is the inner cutoff radius of the dislocation stress field, *r_out_* = 1000 *r_in_* is the outer cutoff radius of the dislocation stress field, and $E_{m}^{\infty }$ and $E_{p}^{\infty }$ refer to the energy per unit length of dislocations in an infinite medium. According to the reference, $\frac{E_{m}^{\infty }}{E_{m}^{\infty }}$ = 0.62. According to the above formula, the contribution of each strengthening mechanism to the yield strength under different cooling processes could be calculated. From 0.5 h to 2.5 h, the contribution of the strengthening mechanism to the yield strength were 117 MP, 107 MP, 102 MP, and 89 MPa, respectively.

When the tempering time was 0.5 h, these dispersed precipitates provide a strengthening contribution of 117 MPa to the material. When the tempering time was 1 h, the yield strength decreased to 667 MPa, and the tempered bainite underwent recovery and recrystallization, resulting in the softening of the material structure. Although the size change in the precipitated phase was not significant, its density decreased, and the contribution of precipitation strengthening decreased to 107 MPa. After tempering for 2 h, the yield strength further decreased to 537 MPa. The size of precipitates increased, the number density decreased, and the contribution of precipitation strengthening further decreased to 102 MPa. The recovery and recrystallization process were completed, and the lath bainite transformed into equiaxed ferrite grains. At 2.5 h, the changes in yield strength and elongation were not significant, and the size of precipitates continued to increase, while the density further decreased. The contribution of precipitation strengthening was adjusted to 89 MPa.

### 4.2. Influence of Heat Treatment on Cu Precipitation

As a result, it was found that with the increase in the tempering time, Cu atoms aggregated in the matrix through the atomic diffusion mechanism, forming large-sized precipitates. This led to the gradual increase in the existing precipitated particles, while the formation rate of the new precipitated particles decreased, resulting in a decrease in the density of particles. As the heat treatment time increased, more Cu atoms continued to diffuse and join the formed precipitate phase, and these precipitated particles further grew. During this process, the strength of the material decreased, and the overall toughness of the material improved.

In addition, unlike previous heat treatments or aging treatments, under the conditions of this experiment, it was found that 9R first transformed into a 3R structure and finally transformed into an FCC structure. The stable Cu precipitation structure was FCC, maintaining a K-S orientation relationship $\left(1\bar{1}1\right)$_FCC Cu_//(0 1 1) _α_, $\;\left[\bar{1}10\right]$_FCC C_//$\left[1\bar{1}1\right]$ _α_. Therefore, it can be concluded that the crystal structure changes in the rich Cu phase during the precipitation process follow the following sequence: B2 → 9R → 3R → FCT → FCC.

## 5. Conclusions

This study analyzed the mechanical properties and microstructure of experimental steel under different heat treatments. The size, morphology, and distribution of the precipitated particles were observed using transmission electron microscopy. The contribution of the precipitation-strengthening mechanism in yielding strength was calculated. The results are as follows:

1. As the heat treatment time increased, the strength gradually decreased and then remained stable, and the elongation gradually increased and then remained stable. In the tempering time between 2 and 2.5 h, the precipitation-strengthening effect and the tempering-softening effect reached an equilibrium state.
2. The microstructure of experimental steel treated at different times was mainly composed of tempered bainite. As the tempering time increased, the size of precipitates increased, and the density decreased. By increasing the heat treatment time, the size and density of the precipitates could be controlled, thereby improving the mechanical properties.
3. This study used the Russell–Brown model to calculate the precipitation-strengthening effect and assessed the contributions of each strengthening mechanism to the yield strength under different cooling processes, which were 117, 107, 102, and 89 MPa, respectively.
4. During the tempering process, nano-Cu precipitates formed in the experimental steel. As the annealing time increased, the precipitates grew and formed a stable 3R-Cu structure.

## Figures and Tables

**Table 1:** Chemical composition of the experimental steel in mass%.

Fe	C	Cu	Ni	Mn	Cr	Si	Mo	V	Al
Bal.	0.02	1.52	1.54	1.0	0.58	0.33	0.29	0.03	0.05

## Data Availability

The original contributions presented in the study are included in the article, further inquiries can be directed to the corresponding author.
